# Member Announcements

**DOI:** 10.3109/03009734.2011.554719

**Published:** 2011-02-11

**Authors:** Torbjörn Karlsson, Anna Rask-Andersen

Dear Members,

We have two forthcoming events that we would like to highlight this time. The first is this year's Martin Holmdahl lecture that will take place on March 15 at 6.00 PM in the Rudbeck laboratory. The Lecture Hall will be announced at a later occasion. The speaker is Torsten E. Gordh and the title of his presentation “A walk on the Via Dolorosa”. This event is a joint project between Selanderska stiftelsen, Uppsala Medicinhistoriska förening, Uppsala Medicinska Seniorer and the Upsala Medical Society.

It is also time for the tradititonal spring meeting of our society. This year we will visit the new “Pedagogicum” at Blåsenhus. Under the guidance of Mia Lindegren we will learn more about “Education in progress” comprising a visit to the “Learning lab” and other facilities. It will all take place on April 5 (for further details, see separate announcement and our web page). The address of the Learning lab is as follows: Kraemers alle' 1A, plan 2 (infart från Norbyvägen vid Botaniska trädgården). Refreshments will be served and your participation has not to be notified in advance.

At this time of the year all members of our society have the privilege of nominating candidates for the Rudbeck prize. The name of the candidate and a short justification has to be sent to the chairman of the society but no later than March 1.

As in our previous member announcements in this journal we would like to highlight our new web page at www.upsalalakareforening.se. We try to keep it updated as much as possible for your convenience. At this site you will find further details on our activities, on how to become a new member and subscriber of this journal, “Veckoprogrammet” and the statutes of the society. Further suggestions on how to improve our web site are welcomed and so are viewpoints on how these announcements should be designed. For instance, should we use the Swedish language?

